# Exploring Radiomics for Classification of Supraglottic Tumors: A Pilot Study in a Tertiary Care Center

**DOI:** 10.1007/s12070-022-03239-2

**Published:** 2022-11-24

**Authors:** Divya Rao, Prakashini Koteshwara, Rohit Singh, Vijayananda Jagannatha

**Affiliations:** 1 Department of Information and Communication Technology, Manipal Institute of Technology, Manipal Academy of Higher Education, Manipal, 576104 India; 2 Department of Otorhinolaryngology, Kasturba Medical College, Manipal Academy of Higher Education, Manipal, 576104 India; 3 Department of Radiodiagnosis and Imaging, Kasturba Medical College, Manipal Academy of Higher Education, Manipal, 576104 India; 4 Data Science and Artificial Intelligence, Philips, Bangalore, 560045 India

**Keywords:** Laryngeal cancer, Computed tomography, Artificial intelligence, Radiomics

## Abstract

Accurate classification of laryngeal cancer is a critical step for diagnosis and appropriate treatment. Radiomics is a rapidly advancing field in medical image processing that uses various algorithms to extract many quantitative features from radiological images. The high dimensional features extracted tend to cause overfitting and increase the complexity of the classification model. Thereby, feature selection plays an integral part in selecting relevant features for the classification problem. In this study, we explore the predictive capabilities of radiomics on Computed Tomography (CT) images with the incidence of laryngeal cancer to predict the histopathological grade and T stage of the tumour. Working with a pilot dataset of 20 images, an experienced radiologist carefully annotated the supraglottic lesions in the three-dimensional plane. Over 280 radiomic features that quantify the shape, intensity and texture were extracted from each image. Machine learning classifiers were built and tested to predict the stage and grade of the malignant tumour based on the calculated radiomic features. To investigate if radiomic features extracted from CT images can be used for the classification of laryngeal tumours. Out of 280 features extracted from every image in the dataset, it was found that 24 features are potential classifiers of laryngeal tumour stage and 12 radiomic features are good classifiers of histopathological grade of the laryngeal tumor. The novelty of this paper lies in the ability to create these classifiers before the surgical biopsy procedure, giving the clinician valuable, timely information.

## Introduction

**Fig. 1:**
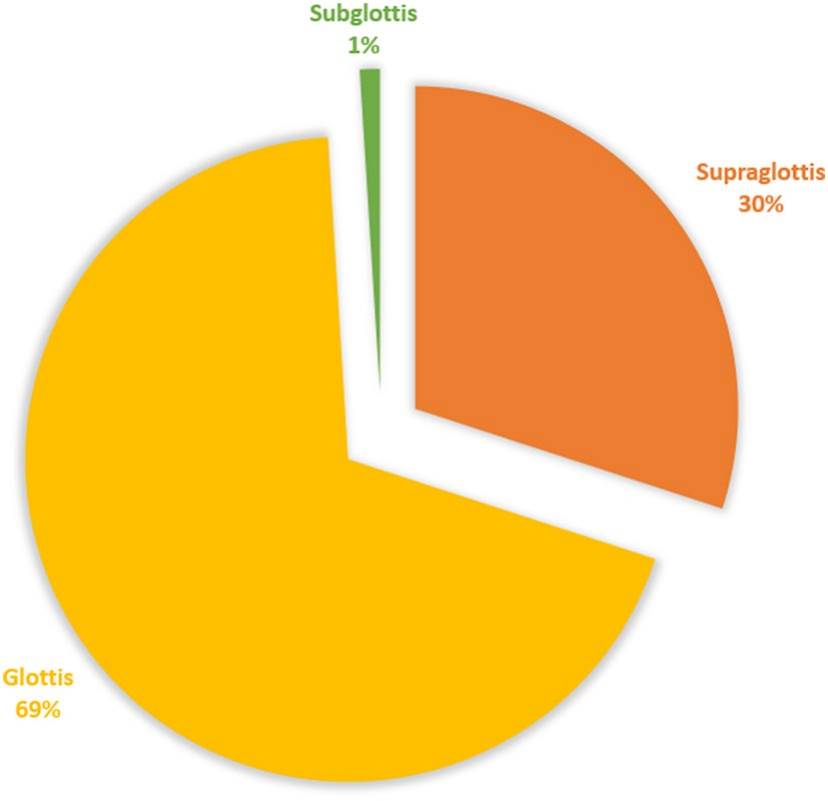
Incidence of laryngeal cancer across subsites

Carcinoma of the larynx is one of the most common forms of head and neck cancer and the 20th most common form of cancer overall [1]. Laryngeal cancer is caused by the spread of malignant cells in the tissues of the voice box or the larynx. The larynx has three main subsites: the supraglottis, the glottis and the sub-glottis. Common sites of the laryngeal cancer origin are the supraglottis and the glottis. Subglottic laryngeal cancers are very rare. Fig 1 shows the incidence of cancer across subsites of the larynx. A significant portion of the population is affected by laryngeal cancer, with over 184,000 new cases detected every year [2].

Computed Tomography (CT) is a radiographic imaging technique that uses a con-trast agent to acquire detailed medical images of the tissue and lesion in the larynx. CT scans are proven to be accurate, time-efficient, and quick in acquisition and computing times and are, therefore, an effective tool for diagnosing laryngeal cancer [3]. CT scans are routinely used for cancer diagnosis in India [1]. Once acquired, the radiologist analyses the CT images for the extent of the tumor and inspects for its spread into adjacent subsites and organs.

Radiomics is a rapidly advancing field that deals with the extraction of quantitative information in radiological images [4]. Radiomic features capture characteristics such as heterogeneity, shape, and texture of lesions or tissues present in the medical images. This information is often combined with other attributes for decision support analysis [5]. Radiologists can visually analyse only a finite number of features via training and experience. However, a lot of contextual information is contained in the pixels of the medial images that can be extracted and analysed [6]. This radiomic data from the medical images can be extracted and mined. Tremendous efforts are being put into discovering previously unknown markers and patterns of disease evolution, progression, and treatment response [7].

**Fig. 2:**
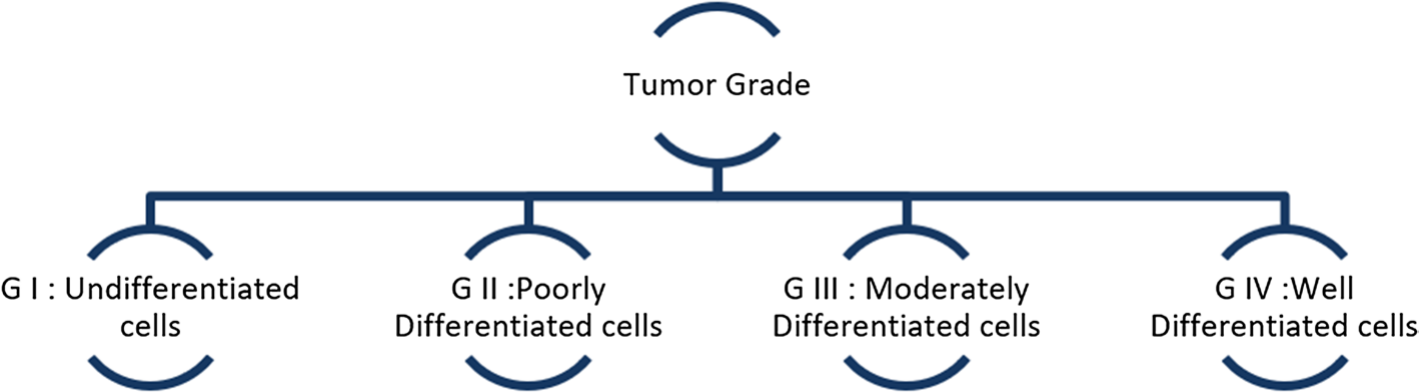
Histopathologic classification of tumor grade

**Fig. 3:**
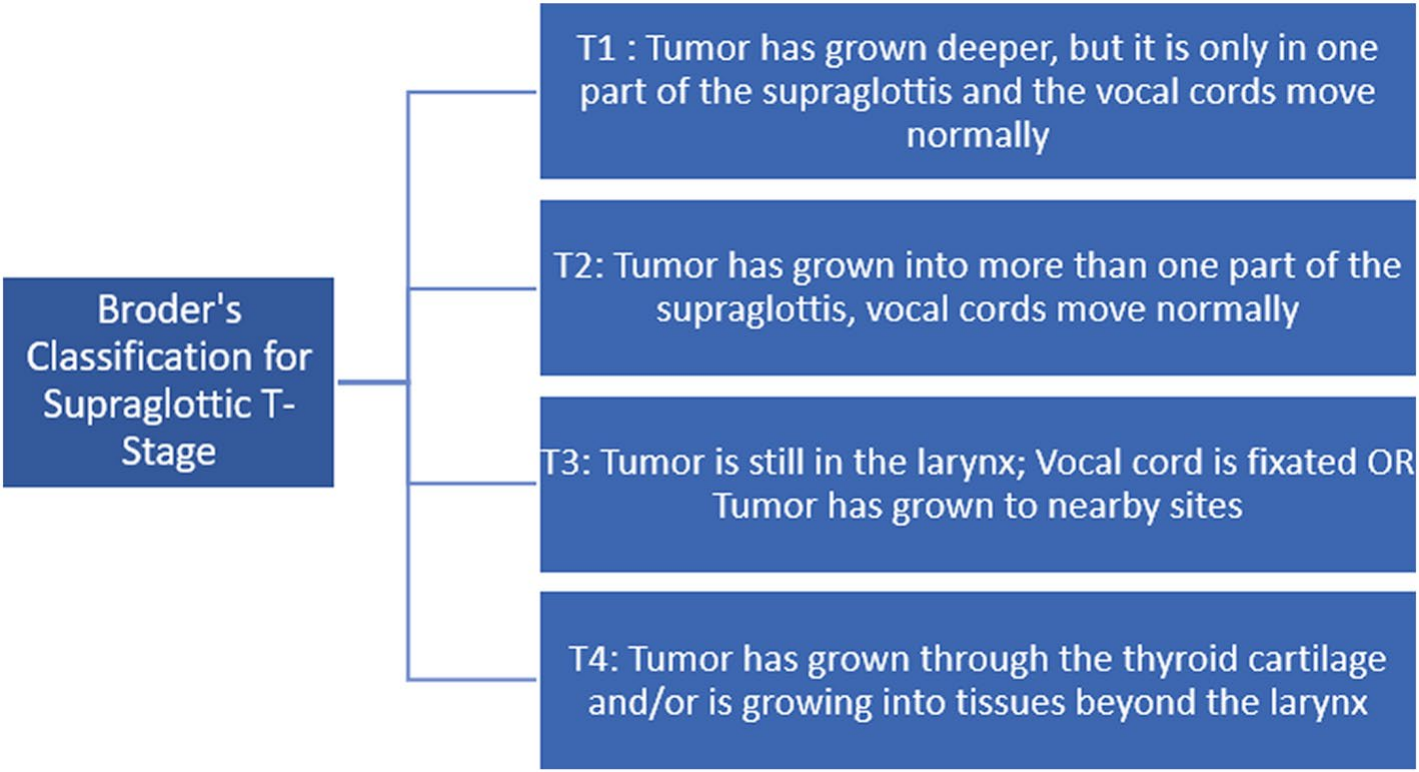
AJCC’s supraglottic cancer T stage classification

Once the Radiographic Imaging is complete (CT/MRI), a tissue sample of the lesion is surgically removed for a histopathology examination. This reveals tumor characteristics such as grade and stage. Tumor grade describes the abnormality of the tumor cells under a microscope [6. High-grade cells are well differentiated abnormal cells that have rapid growth. Lower-grade tumors are poorly differentiated and multi-ply faster than normal cells but not as much as high-grade tumor cells. The grades of laryngeal tumor have been illustrated in Fig. 2. The TNM staging system followed by the AJCC uses numbers to describe the cancer [8]. The T stands for Tumor, and the number following it represents the size of the tumor. N stands for Node, and the number describes the spread of cancer to the nodes. M indicates if the tumor metastasized [8]. The T stages and their relevance have been illustrated in Fig. 3 below.

## Literature Survey

This section comprises a brief literature survey of how radiomics has been explored in laryngeal cancer.

As application of radiomics to laryngeal cancer is an emerging area of research interest, there is limited literature available. AD Kumar et al. [9] reviewed all studies (n = 15) that applied radiomics to laryngeal cancer. In the review, two studies assisted the prediction of cancer stage using radiomics. Estimation of patient survival time and prediction of prognostic factors such as treatment response were incorporated. The details of accuracy were not mentioned.

Guo et al. [10] used radiomic features computed from CT images of the larynx and investigated the accuracy of predicting thyroid cartilage invasion. They had a dataset of 265 CT scans. Using an oversampling method, they balanced their dataset and used Logistic Regression to obtain an area under the ROC curve of 0.905. They claimed that their model predicted cartilage invasion significantly better than the radiologist assessment. Radiomics can be used as a non-invasive alternative to the preoperative prediction of thyroid cartilage abnormality.

Agarwal et al. [11] worked with radiomic features of a dataset of 60 individuals analyzed after chemotherapy treatment and a follow-up period of 24 months. They concluded that Medium texture entropy, a radiomic feature, can be used as an accurate predictor for inferior local control. It could also predict survival where laryngectomy would not be necessary and also in laryngopharyngeal cancers where the stages were advanced; this feature could be used to complement clinical and radiological findings to determine prognostics. A filtration-histogram technique was used where the filtration step extracted and enhanced features of different sizes and intensity variations corresponding to a particular spatial scale filter. The ability of texture analysis to predict LFS or local control was determined using Kaplan-Meier analysis and the multivariate cox model.

Wu et al. [12], in their comprehensive multicentric study, used 211 laryngeal images for the problem of stage prediction into T3/T4. Using radiomics, peritumoral models were constructed and observed the influence of the peritumors (compared intra-tumors vs. intra-peritumors) for stage prediction. The conclusion of the study was that the dataset that used radiomic feature assessment(intra-peritumors) performed better 0.660 versus 0.579 (*P*-value: 0.431).

## Materials and Methods

**Fig. 4:**
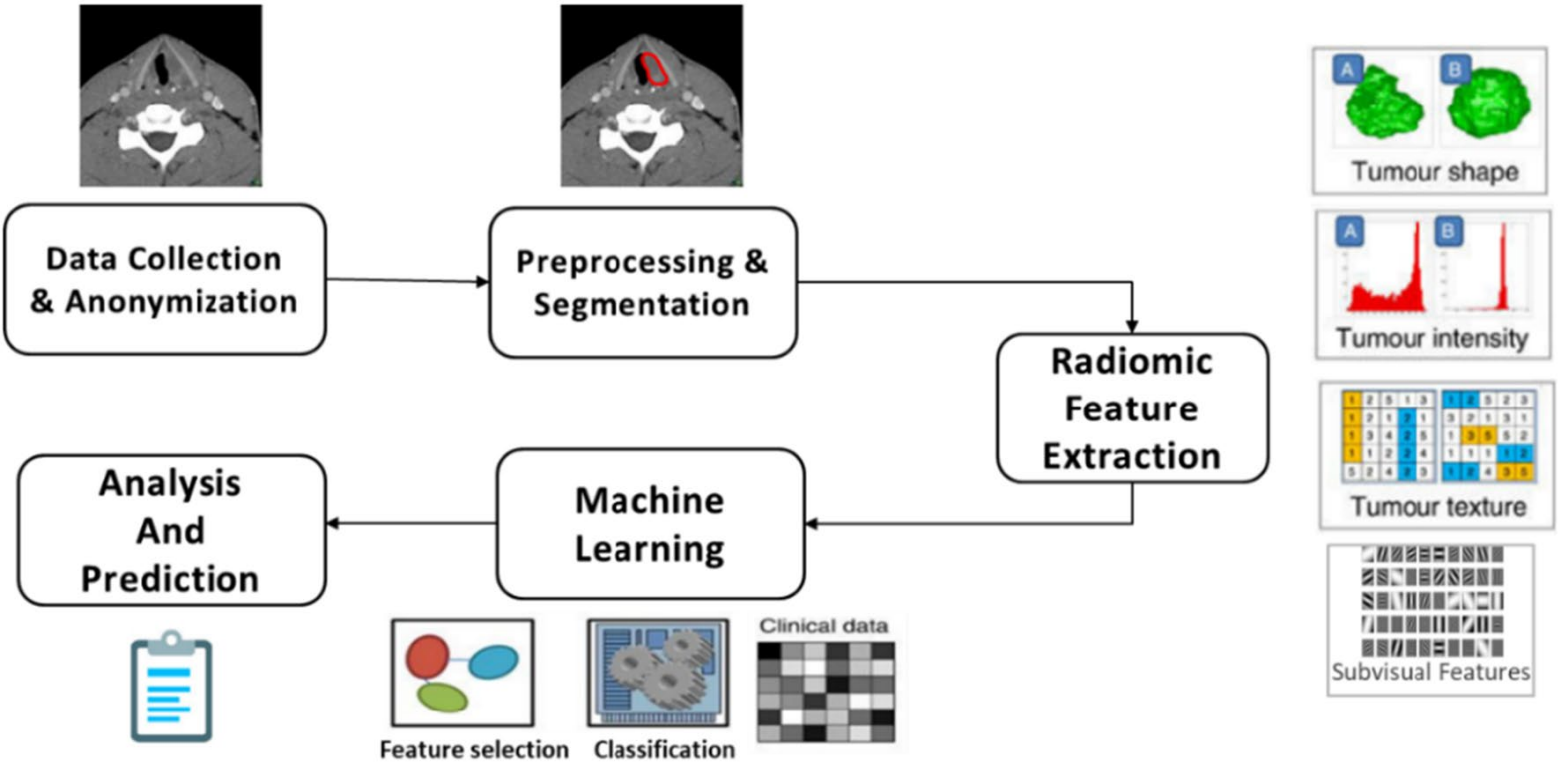
Workflow of radiomic feature extraction

An initial round of suitable data identification and collection followed by which this data was converted to the required format and preprocessed. The radiological images and the obtained expert segmentation masks were used to obtain 280 radiomic features per image. The features were then examined for the possibility of prediction of our variables of grade and stage of the laryngeal tumor. The steps are explained in detail below. The methodology followed in the work is illustrated in Fig. 4.

### Image Acquisition

The first step was identification of patients diagnosed with laryngeal cancer. The CT images of the respective patients were retrieved in the native Digital Imaging and Communications in Medicine (DICOM) [13] format from the Picture Archival and Communication System (PACS) [14]. The histopathology reports, the radiologists’ reports and the final staging information from the discharge summary from the clinician was collected. Twenty contrast CT images that contained lesions in the supraglottis subsite were con-sidered for this study.

### Image Processing and Segmentation

The contrast-enhanced CT images were converted from DICOM to NIfTI (Neu-roimaging Informatics Technology Initiative) format [15]. A window of 60,360 was chosen in the ITK-Snap tool [16] to ensure the contrast of the tumor was enhanced and visible to the radiologist for annotation. An expert radiologist manually annotated all the images slice by slice. The area of presence of tumor tissue was manually annotated for all the slices in all the images considered in our study. This comprised of the segmentation of tumor region step.

### Radiomics Feature Extraction

The next step was to extract the radiomic features present in the segmented area of the images. PyRadiomics [17] was used to extract over 280 features from the segmented images. Histogram features [18], Form factor Features, GLCM features [19], Harlick features, GLSZM features [20], Texture features, Intensity features, Shape features [21, 22] and various filters were applied and the radiomic features were stored in a csv file, made ready to apply the classification algorithms.

### Prediction Engine Model

The radiomic features stored in the file were then used to predict the stage and grade of the tumor. Stages T1 and T2 were grouped as Low Stage Tumors. Stages T3 and T4 were grouped as High Stage Tumors. For the prediction of grade, the two classification classes were Grade II (moderately differentiated squamous cells) and Grade III (poorly differentiated squamous cells). The data was divided into a 80:20 split for training and validation. A support vector machine (SVM) model [23] was built by using features selected using the maximum relevance minimum redundancy (mRMR) algorithm [24]. The mRMR method ranked each feature according to its relevance to the status and redundancy with other features. An SVM score was calculated for each patient to reflect the grade and stage probability from the SVM model [25].

## Results

**Fig. 5:**
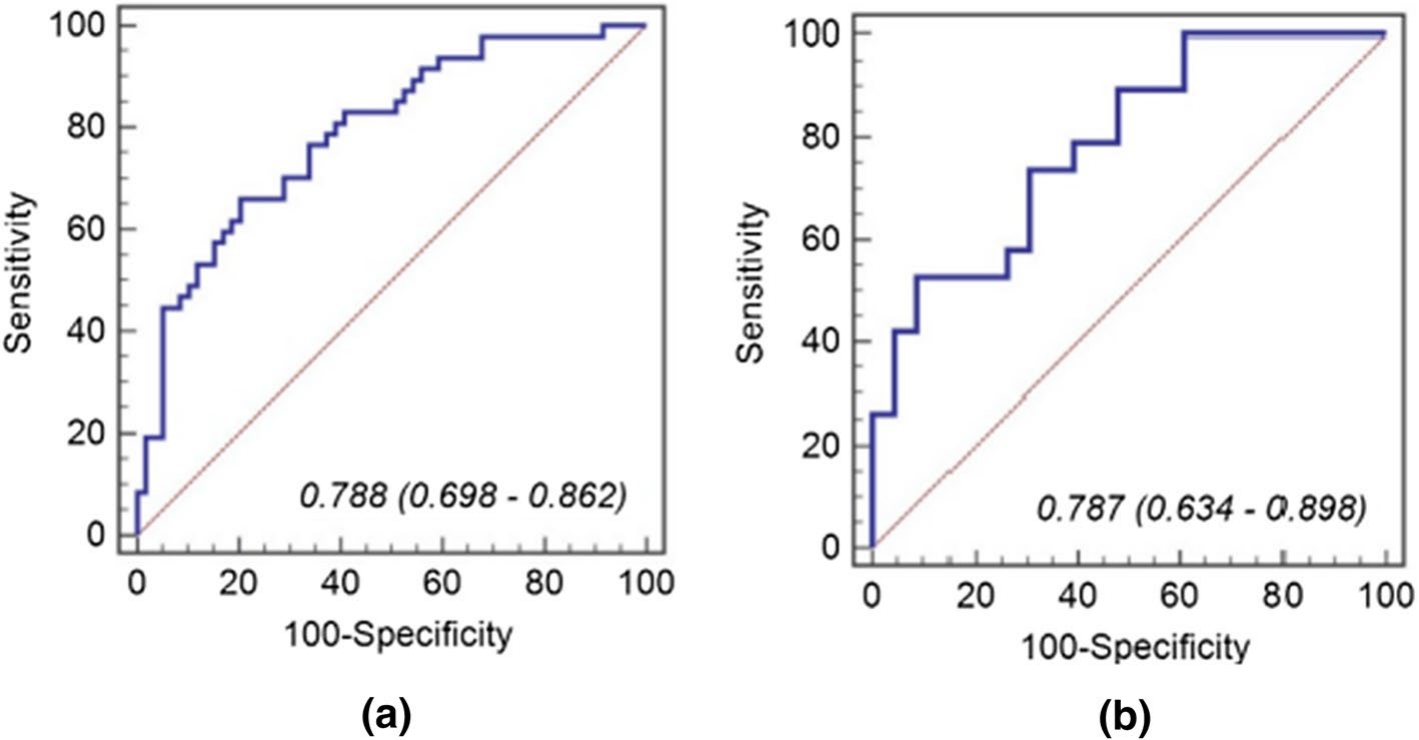
The ROC curves of the SVM model in the training group (**a**) and the validation group (**b**)

A total of 20 patients with supraglottic cancer were included in this study. Each of the contrast CT scan was annotated with an expert radiologist slice by slice and 280 radiomic features were extracted from every image. The stage of carcinoma and histo-pathological grade of the tumors were the variables. Using the machine learning prediction engine model, feature selection was performed on the data. These radiomic features are not visible but the values can computed from the CT images. The threshold was defined as 0.49 based on the Youden Index. Patients with scores higher than the threshold were classified as high stage, while patients with scores lower than the threshold were classified as having low stage supraglottic tumor. A similar method was used to predict the grade. The prediction accuracy was 74.5% for the training group and 69.5% for the validation group. The ROC curves and scatter plots for the SVM score are presented in Fig. 5.

**Fig. 6:**
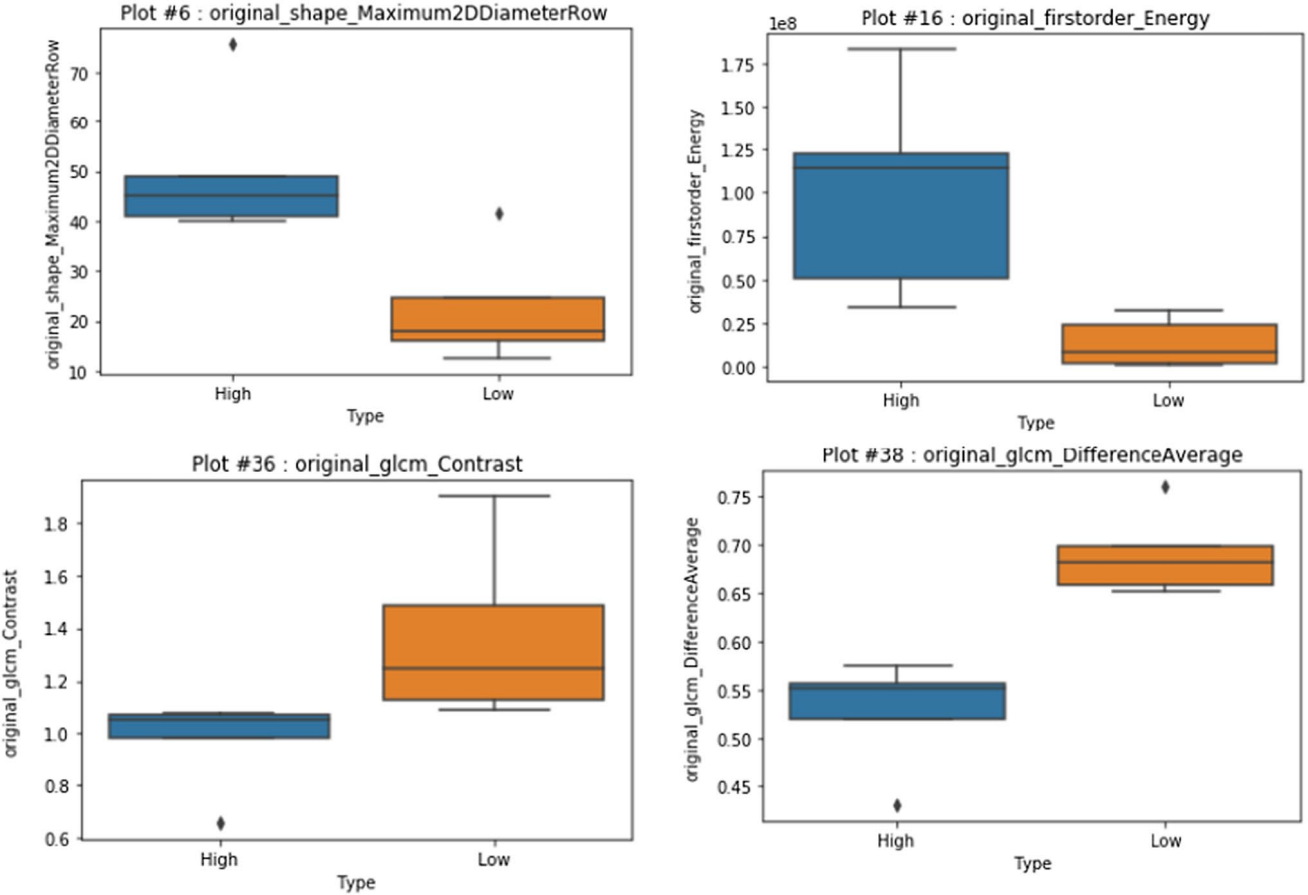
Radiomic features that are good predictors of stage

Table 1 lists the select radiomic features classify the stage of the tumor into high stage or low stage. Figure 6 shows the boxplots of a few of the features in Table 1.

**Table 1.** Radiomic features that can be used to classify the stage of Supraglottic Tumor.

Shape maximum 3D diameter	GLRLM run length non uniformity normalized
Shape maximum 2D diameter row	GLRLM run percentage
First order energy	GLRLM run variance
GLCM contrast	GLRLM short run emphasis
GLCM difference average	GLSZM large area emphasis
GLCM difference entropy	GLSZM large area low gray level
GLCM Id, GLCM Idm	Emphasis
GLDM dependence non uniformity	GLSZM zone percentage
GLDM gray level non uniformity	GLSZM zone variance
GLRLM gray level non uniformity	NGTDM busyness
GLRLM long run emphasis	NGTDM coarseness
NGTDM contrast	NGTDM complexity

**Fig. 7:**
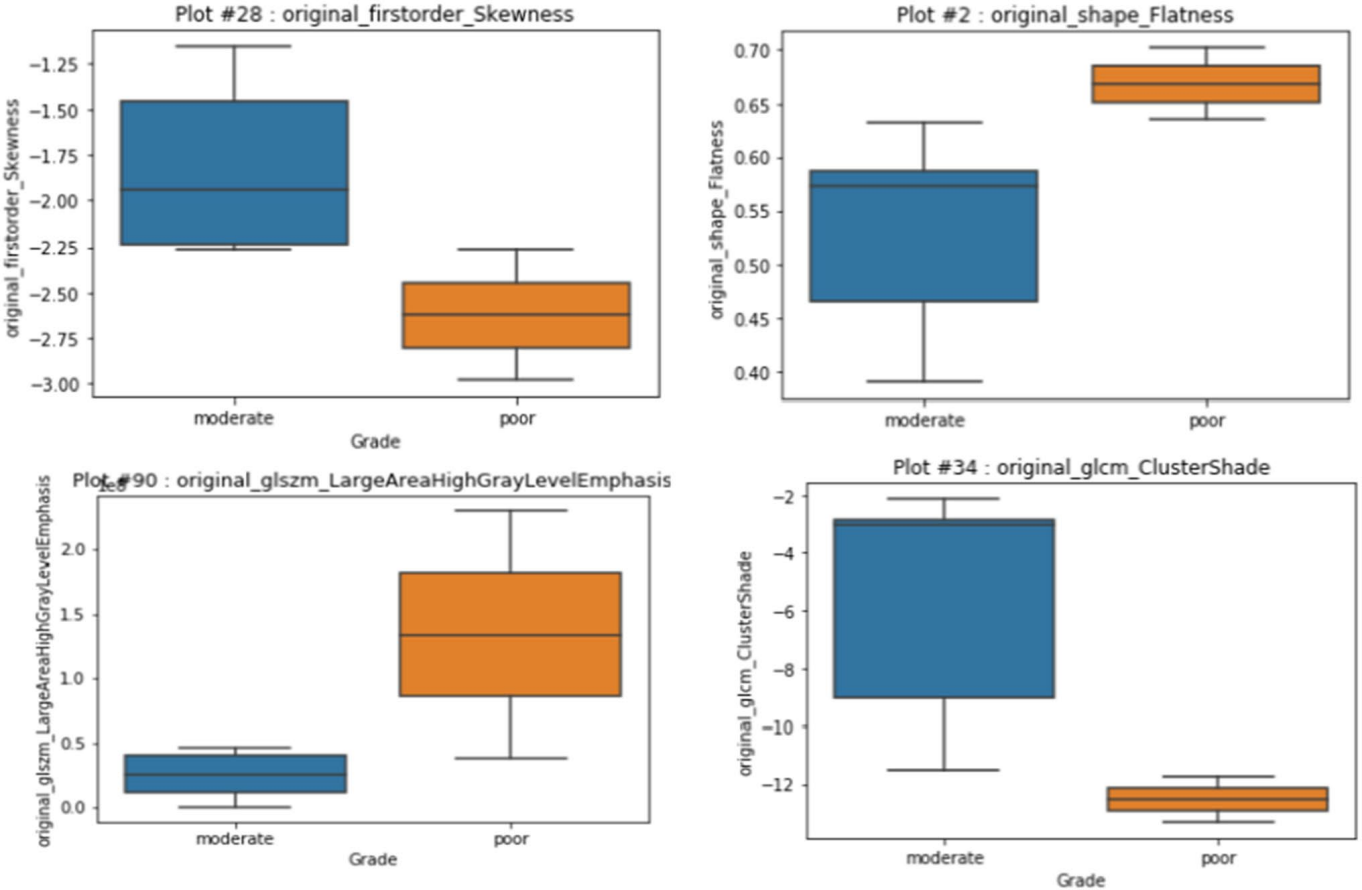
Radiomic features that are good predictors of grade

Likewise, for the classification of the image into tumor grades, our feature prediction engine selected the radiomic features represented in Table 2. These features were found to be useful in predicting the grade of the tumor where Grade II represented the moderately differentiated squamous cells) and Grade III is poorly differentiated squamous cells. Figure 7 shows the boxplots of a few of the features in Table 2.

**Table 2.** Radiomic features that can be used to classify the grade of supraglottic tumor successfully

GLRLM long run high gray level emphasis	GLSZM large area high gray level emphasis
Shape flatness	First order skewness
GLCM cluster shade	

## Conclusion

In this study, 280 features were extracted from 20 CT images with supraglottic laryngeal carcinoma. Feature selection methods determined the specific radiomic features to predict the stage into high and how and grade of the tumor cells into poorly differentiated and well-differentiated. This preliminary analysis is an encouraging result of the ability to create classifiers. The application of this work would be to provide the clinician with valuable information before conducting an invasive surgical biopsy procedure.

The future work of this study will include an increase in the size of the dataset and explore combinations of features for the prediction of prognostic markers of laryngeal cancer. The mining of radiomics data has a promising future. The conclusions drawn are robust when the study sample sizes are big, heterogeneous and multicentric. A unified research effort to standardize the annotation, segmentation and radiomics data computation practices would lead to the translation of results across centres, increasing the overall impact and usefulness.
